# Advancing MRI diagnostic practices in rectal cancer: exploring the impact of web-based multi-reader study participation

**DOI:** 10.1007/s00261-025-05104-6

**Published:** 2025-07-28

**Authors:** Elisabeth P. Goedegebuure, Max J. Lahaye, Nino Bogveradze, Najim El Khababi, Joost J. M. van Griethuysen, Artem Khmelinksii, Monique Maas, Regina G. H. Beets-Tan, Doenja M. J. Lambregts

**Affiliations:** 1 Department of Radiology, The Netherlands Cancer Institute, Amsterdam, The Netherlands; 2 GROW Research Institute for Oncology and Reproduction–University of Maastricht, Maastricht, The Netherlands; 3 Department of Biomedical Imaging and Image-guided therapy, Medical University of Vienna, Vienna, Austria; 4 Department of Radiology, American Hospital Tbilisi, Tbilisi, Georgia; 5 Department of Radiotherapy, University Medical Center Utrecht, Utrecht, The Netherlands; 6 Department of Radiology, University Medical Center Utrecht, Utrecht, The Netherlands; 7 Director of Imaging Innovation Research , The Netherlands Cancer Institute, Amsterdam, The Netherlands

**Keywords:** Magnetic resonance imaging, Rectal neoplasms, Neoplasm staging, Response

## Abstract

**Purpose:**

To explore the impact of participation in web-based validation studies on personal and institutional diagnostic practices for MRI rectal cancer (re)staging.

**Methods:**

An online questionnaire was distributed to radiologists who had participated in one or more previously conducted and published multi-reader validation studies focused on rectal cancer staging and response evaluation. The questionnaire included general questions about the web-based platform used for these studies, as well as study-specific questions addressing the tools under investigation, such as diffusion-weighted imaging (DWI), different MRI response grading systems, and the sigmoid take-off (STO).

**Results:**

Among 25 respondents from 14 countries, 52% reported significant improvements in their personal reporting practice as a result of their study participation; 36% also observed a significant impact on local diagnostic practices in their institution after discussing the study results with their colleagues. Key reported effects included increased use of DWI for restaging, increased use of the STO to discern rectal from sigmoid cancer, enhanced confidence in using diagnostic tools and grading systems, greater adoption of structured reporting templates, and more frequent integration of organ-preserving treatment considerations into radiological assessments. Respondents also emphasized the importance of receiving feedback to maximize the educational benefits of participating in such studies.

**Conclusion:**

Web-based validation studies can positively influence radiologists’ reporting practices, fostering the adoption of novel diagnostic tools through education and collaborative knowledge sharing. Future studies should focus on incorporating consistent feedback mechanisms and integrating specific training modules to maximize the impact of these platforms on clinical practice.

**Supplementary Information:**

The online version contains supplementary material available at 10.1007/s00261-025-05104-6.

## Introduction

Cancer treatment is becoming increasingly personalized to meet the specific needs of each patient, with imaging playing a pivotal role in identifying these needs and advancing personalized medicine. Despite recent advances in artificial intelligence (AI) and quantitative imaging, the visual interpretation of diagnostic images by radiologists remains essential for staging, response evaluation, and treatment planning. To support this process, various diagnostic grading systems and standardized reporting templates have been introduced to improve consistency and diagnostic accuracy for radiologic reporting in oncology. However, before such tools can successfully be implemented in everyday clinical practice their applicability and generalizability will need to be confirmed on a large scale—not just by experts, but also by radiologists in everyday clinical settings. Unfortunately, this important step is often overlooked, creating a barrier to the translation of diagnostic tools from research to routine clinical use.

To address this, our research group developed an online platform and research infrastructure (iScore), which combines an open-source DICOM viewer [1] with customizable electronic scoring forms (eCRFs). This in-house developed webplatform, described in detail in previous publications [2–6], enables radiologists from diverse geographic locations and institutions to collaboratively evaluate imaging data– ideally sourced from multiple centres– to foster knowledge exchange and assess the generalisability of diagnostic grading systems and reporting tools. Recently, we leveraged this platform to conduct several diagnostic validation studies focused on rectal cancer imaging, related to both primary staging and restaging after neoadjuvant treatment. In specific, these previously published works addressed the use of the structured reporting and staging template published by the European Society of Gastrointestinal and Abdominal Radiology (ESGAR) [2, 7], the sigmoid take-off (STO) as a landmark to discern rectal from sigmoid cancer [3], and evaluated various grading systems to predict and monitor rectal tumor response [4–6], such as the MRI tumor regression grade (mrTRG), ‘split scar sign’ and different DWI-based scoring systems [8–11].These tools can aid in the initial staging and treatment stratification of rectal cancer [12], and to evaluate response and guide further management after neoadjuvant treatment. In the latter setting, restaging MRI can guide surgical management, but also– when combined with endoscopy– help identify patients with a clinically complete (or near-complete) response [13]. For these patients, various (expert) centers around the world nowadays offer the option of organ-preservation (“watch and wait”) as an alternative to radical resection. Radiologists are central to this process, influencing multidisciplinary team (MDT) discussions and treatment decisions at both initial staging and restaging.

In our previously published multi-reader validation studies, a total group of over 30 radiologists from 18 countries participated. These radiologists collaboratively evaluated more than 400 MRIs using study-specific eCRFs embedded in the online platform, and receiving feedback to their scorings after completing the cases. Diagnostic outcomes of these studies have been addressed in the previous publications [2–6]. The goal of our current study was to explore the potential impact of participating in these web-based studies on the reporting habits and local institutional workflows of the radiologists involved. To achieve this, we distributed an online questionnaire to radiologists who took part in our previous multi-reader projects, aiming to gather insights into their experiences, learning outcomes, and the degree to which their involvement influenced both their personal reporting practices and clinical workflows at their home institutions. This paper presents the findings from that questionnaire.

## Materials and methods

This study concerns a questionnaire-based survey for which no patient or imaging data were collected or analyzed. In accordance with institutional and ethical guidelines, formal approval from an Institutional Review Board (IRB) and informed consent were not required.

### Target group

The target group for this study included all radiologists who participated as study readers in one or more of our previously mentioned multi-reader validation studies focused on primary rectal cancer staging using standardized reporting templates [2], use of the sigmoid take-off to distinguish rectal from sigmoid cancer [3], and various grading systems to predict and monitor response to neoadjuvant treatment [4–6]. Further details of these studies (including use of patient and imaging data, methods of analysis, specifics on eCRFs and use of the webplatform, and diagnostic outcomes) can be found in the original publications [2–6]. Radiologists who only participated in these studies as the expert standard of reference, or radiologists who were involved in the development of the grading systems/methods under evaluation, were not included in the target group.

### Online questionnaire

Radiologists from the target group were invited to complete an online questionnaire developed by the authors using Google Forms. The questionnaire included 39 question: 13 general questions for all participants and a total of 26 study specific questions directed only to those who had participated in the respective studies. The general questions aimed to establish the participants’ clinical backgrounds, collect feedback on the use of the online research platform (iScore), and explore how study participation– including case reading, feedback received, and review of the final results– impacted their personal reporting practices and local workflows at their home institutions. The study-specific questions focused on the effects of individual study projects, covering topics such as the use of the STO, mrTRG and modified mrTRG, DWI response methods, the split scar sign, and standardized reporting templates [2–6]. A more detailed glossary of the reporting tools and grading systems addressed in the questionnaire is provided in *Supplement 1*. Most questions were multiple-choice, with several employing 5-point Likert scales to measure experience levels (e.g., “Did participation effect your reporting practice” with options ranging from “not at all” to “extremely”). Participants were invited to provide free-text comments to elaborate on their answers. The full questionnaire is provided in Supplement 2.

### Data analysis

**Fig. 1:**
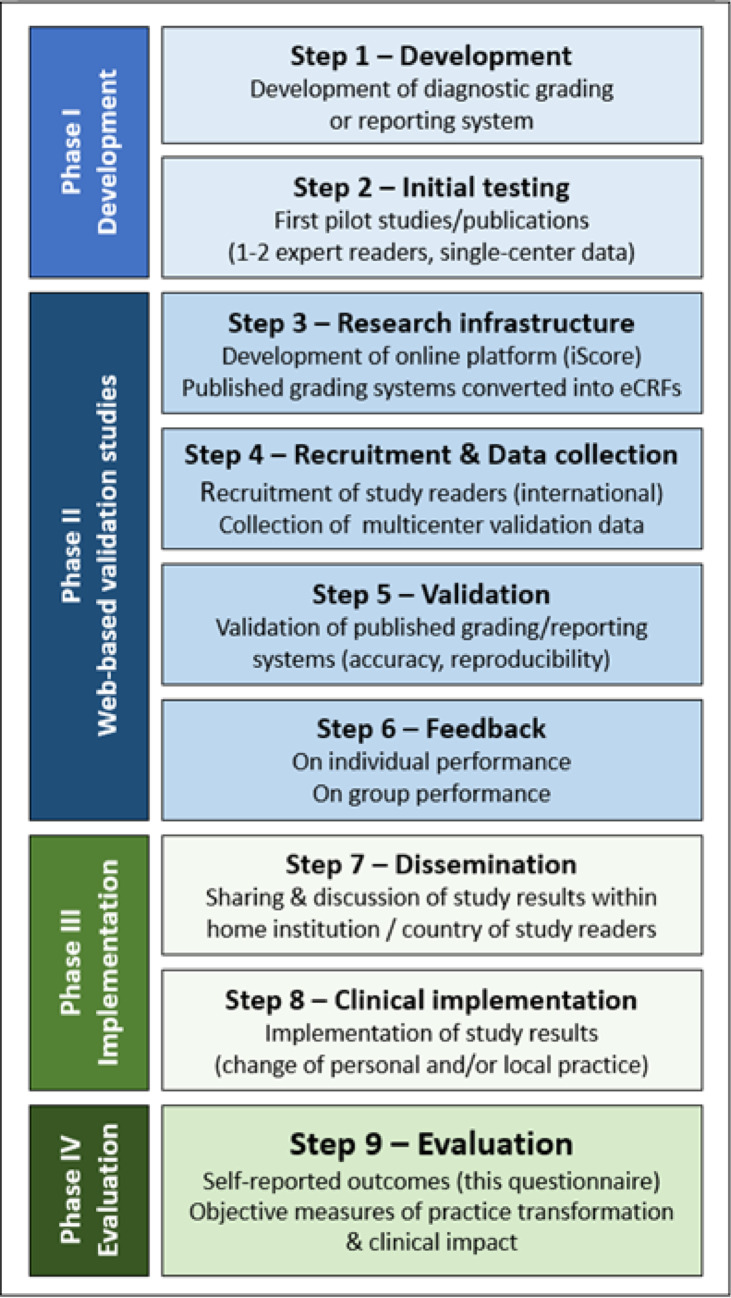
Overview of study steps

The questionnaire results were analyzed using descriptive statistics. Figure 1 illustrates the progression from the initial development and publication of new diagnostic grading systems through to their validation and clinical implementation, highlighting the steps that informed the questions addressed in this study.

## Results

### Respondents’ demographics

The questionnaire was sent to 35 radiologists from 18 countries; twenty-five completed it, accounting for a response rate of 71%. The majority of the 25 respondents (80%) were abdominal radiologists, half of whom had dedicated expertise in (colo)rectal cancer. Among the ten non-responders, 40% were male (compared to 32% in the group that completed the survey). These non-responders originated from 6 different countries, of which the majority were represented by the radiologists who completed the survey. Further details for the 25 radiologists who completed the survey are provided in Table 1.

**Table 1:** Main characteristics of the 25 respondents

Respondents	Total	*N* = 25	% (100%)
Sex	Male	8	32
	Female	17	68
Profession	Abdominal radiologist	20	80
	With dedicated expertise in (colo)rectal cancer	10	40
	General radiologist	3	12
	Resident	2	8
Workplace	Comprehensive cancer center	6	24
	Academic hospital	9	36
	General hospital	8	32
	Private practice/other	2	8
Country of origin	United Kingdom	5	20
	The Netherlands	3	12
	Italy	3	12
	Romania	2	8
	Switzerland	2	8
	India	2	8
	Brazil	1	4
	Denmark	1	4
	Georgia	1	4
	Chile	1	4
	Belgium	1	4
	Sweden	1	4
	Germany	1	4
	United States	1	4

### General outcomes

**Fig. 2:**
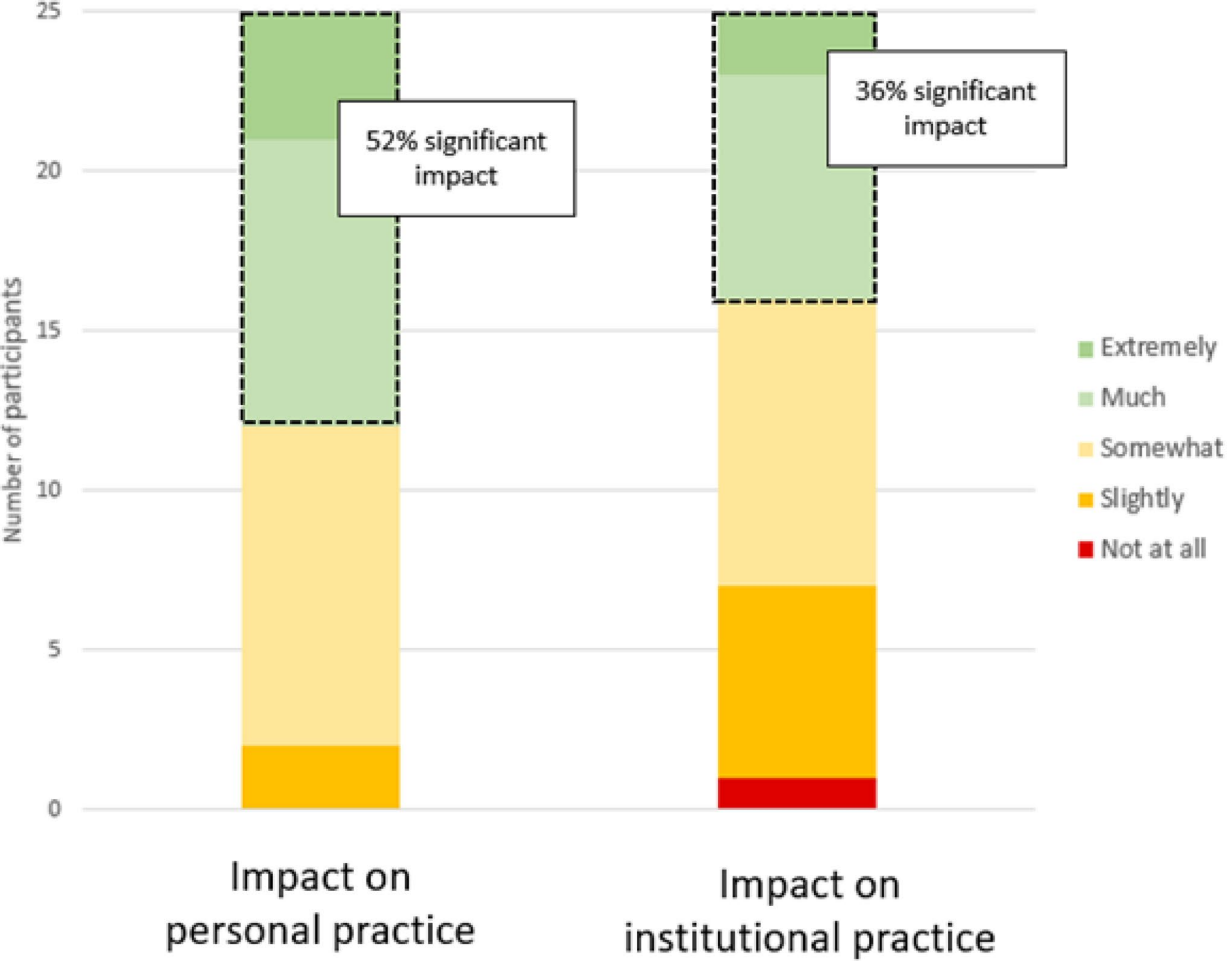
Distribution of reported effects on personal and institutional reporting practice

Figure 2 provides a graphical overview of the overall reported impact on respondents’ personal and local reporting practices. 52% of respondents indicated that participating in the studies had a significant effect (rated as “much” or “extremely”) on their own reporting habits, while an additional 40% noted a moderate impact. Regarding local practice at their home institutions, 36% reported a significant effect after discussing the study results with colleagues, with another 36% reporting a moderate effect. When asked to elaborate, 76% of respondents said they shared the study results with their clinical peers, and within this group, 53% actively engaged in discussions about applying the results to improve local practice.

Table 2 summarizes the most commonly reported comments from respondents, describing how participation in the studies influenced their personal and/or institutional practices.

**Table 2:** Most frequently provided comments on study participation

*Effect on personal practice*
Increased confidence in staging and reporting
Reinforced confidence in the benefits of specific tools (e.g., DWI)
Improved reporting structure, leading to more systematic and structured reports
Learning curve effect by evaluating a large number of rectal cancer cases in a short time
Increased familiarity with applying specific methods and tools (e.g., sigmoid take-off, DWI patterns)
*Effect on local practice (at home institution)*
Knowledge shared with colleagues and implemented in practice
Enhanced collaboration between radiologists and clinicians, including better management of expectation regarding MRI’s diagnostic capabilities
Diagnostic confidence levels are more frequently reported and used to guide clinical-decision making during MDT discussions

### Feedback on research platform and study infrastructure

The majority of respondents (96%) rated their overall experience with our online research platform as either highly positive (68%) or moderately positive (28%). When asked to elaborate on their experiences, most participants found the platform intuitive and easy to use. Regarding feedback on their case readings, 44% of respondents reported receiving general or detailed feedback during or after their participation in the studies, 16% could not recall whether they received any feedback, and 40% indicated they did not receive any detailed feedback beyond the final manuscript presenting the study results. Among those who received feedback, the majority (82%) found it highly useful– 27% rated it as extremely useful, 55% as very useful, 9% as moderately useful, and 9% as slightly useful. Overall, 24% of respondents expressed a desire for more personalized feedback, or general feedback highlighting the most common and significant errors made.

### Study specific outcomes

An overview of the main outcomes retrieved from the respective study-specific sub-questionnaires is provided in Table 3. Key effects included a marked increase in the use of sigmoid take-off, more frequent use of DWI for response assessment and restaging, and greater adoption of structured reporting templates for baseline staging– primarily the reporting template published by the European Society of Gastrointestinal and Abdominal Radiology (ESGAR) [7]. In addition, respondents indicated that they more commonly included a response estimation and/or anticipated the chance that a patient might achieve organ preservation at the time of baseline staging to better inform MDT discussions.

Between 76 and 100% of respondents, depending on the individual study, agreed that radiological guidelines should be updated to reflect the study findings. Specifically, participants involved in restaging and response evaluation studies recommended incorporating a standardized response grading system– preferably including DWI– into the structured reporting template for restaging. Those involved in the STO study expressed that baseline staging reporting templates should routinely include a description of tumor location relative to the STO, based on the study outcomes.

**Table 3:** Study specific responses regarding the use of different MRI tools, grading and reporting systems

	*Before* participation in study	*After* participation in study
*Questions related to baseline staging*		
Do you use the sigmoid take-off as a landmark to discern rectal from sigmoid cancer?^a^	100% no	0% no
	0% occasionally	20% occasionally
	0% sometimes	0% sometimes
	0% most of the time	20% most of the time
	0% always	60% always
Do you use a standardized (pro forma) template to report rectal MRI?^b^	12% no	6% no
	29% yes, institutional template	6% yes, institutional template
	41% yes, ESGAR template	65% yes, ESGAR template
	18% yes, alternative template	23% yes, alternative template
Do you estimate the chance that a tumor will show a good response to treatment as part of your report (i.e. to guide MDT discussions on organ-preservation)?^c^	92% no	54% no
	0% occasionally	31% occasionally
	8% sometimes	8% sometimes
	0% most of the time	8% most of the time
	0% always	0% always
*Questions related to restaging and response assessment after neoadjuvant treatment*		
Do you use the mrTRG for restaging and response evaluation?^c^	23% no	31% no
	8% occasionally	8% occasionally
	23% sometimes	0% sometimes
	15% most of the time	23% most of the time
	31% always	38% always
Do you use DWI for restaging and response evaluation?^c^	0% no	0% no
	15% occasionally	8% occasionally
	0% sometimes	0% sometimes
	23% most of the time	8% most of the time
	62% always	83% always
Do you use the split scar sign for restaging and response evaluation?^c^	54% no	15% no
	31% occasionally	31% occasionally
	8% sometimes	31% sometimes
	0% most of the time	8% most of the time
	8% always	15% always
Do you report the yT-stage as part of you restaging and response evaluation?^c^	8% no	15% no
	8% occasionally	8% occasionally
	8% sometimes	0% sometimes
	15% most of the time	15% most of the time
	62% always	62% always

*ESGAR* European Society of Gastrointestinal and Abdominal Radiology

^a^ based on questionnaire responses of *n* = 5 respondents who participated in study by Bogveradze et al. [3]

^b^ based on questionnaire responses of *n* = 17 respondents who participated in study by El Khababi et al. [2]

^c^ based on questionnaire responses of *n* = 13 respondents who participated in studies by El Khababi et al. [4–6]

## Discussion

When asked to reflect on their experiences via an online questionnaire, 52% of radiologists from an international group participating in online multi-reader MRI studies for rectal cancer (re)staging reported that their involvement significantly influenced their personal reporting practices. Additionally, 36% noted a significant effect on local clinical practice within their departments after discussing the study outcomes with their colleagues. Although this questionnaire is descriptive and does not provide an objective measure of clinical impact, these findings highlight the potential of web-based validation studies to promote evidence-based practice and facilitate knowledge dissemination within radiology departments.

Our results suggest that participation in research validating diagnostic tools can influence clinical practice through various mechanisms. Respondents reported that repeatedly applying new methods to an external dataset stimulates a strong learning effect. This aligns with findings by El Khababi et al. who showed that virtual case-based training for rectal cancer staging improves diagnostic accuracy and confidence, serving as an effective alternative to in-person training [14]. Using web-based platforms in a research setting replicates such training environments by providing access to a diverse set of cases and incorporating generalized or personalized feedback mechanisms. This was also reported by several survey respondents, who noted an increased diagnostic confidence in (re)staging rectal cancer and in applying the diagnostic tools under evaluation after they participated as study reader. Web-based validation studies can be strong tools to foster participation-based learning by embedding radiologists within dynamic, practice-oriented learning environments. This aligns with Lave and Wenger’s theory of ‘situated learning’, which posits that knowledge is most effectively acquired through active participation in real-world activities within a ‘community of practice’ [15]. In the context of digital radiology research and education, the web-based platforms used in our studies can be seen as such communities– networks where shared diagnostic challenges drive collective learning, peer comparison and reflective practice [16]. As radiology increasingly shifts toward digital collaboration and remote education, integrating principles from social learning into study designs could further enhance their educational value and clinical impact. 

Participants also reported increased use of specific tools studied, such as DWI for restaging and the sigmoid take-off (STO) as a landmark to discern rectal form sigmoid cancer. Many previous studies have shown that adding DWI to standard rectal MRI improves restaging accuracy, particularly in identifying complete responders after neoadjuvant treatment [17, 18]. Reflecting this, recent ESGAR and Society of Abdominal Radiology (SAR) guidelines recommend routine DWI use to distinguish residual tumor from complete response in the restaging setting [7, 19]. In the previously published multi-reader publication addressing the use of DWI in comparison to other response methods (mrTRG, split scar), DWI-based methods showed the most favorable overall results, in particular in terms of interreader agreement and specificity. Also, readers who participated in that study preferred the use of DWI over other methods [5]. Notably, prior to their study participation, 38% of respondents did not yet routinely use DWI, a figure that dropped to 17% after participation, with many reporting increased confidence in using DWI for restaging.

The STO was first reported as a preferred landmark by international consensus in 2019 [20]. While included in some guidelines (e.g., Dutch national guidelines [21]), it was not widely adopted when Bogveradze et al. launched their study addressing the STO in a multi-reader setting in 2022 [3]. Results of that study—which included 11 radiologists– showed that consensus on how to discern rectal from sigmoid tumors using the STO could be reached in 63% of cases, when applying a dichotomous classification. According to our questionnaire results, none of the participants had used the STO before they participated in the study, but after reviewing 155 cases and reading the study results, 80% of respondents reported using the STO routinely or most of the time. These changes in DWI and STO use highlight the potential of large-scale validation studies in bridging the gap between research and clinical practice, accelerate guideline adoption, and promote evidence-based practice in radiology.

Another important impact reported was the increased awareness and inclusion of organ-preserving treatment considerations in radiological reports and multidisciplinary team (MDT) discussions. Recently, there has been a paradigm shift towards offering non-operative management (‘watch-and-wait’) or local excision for patients who show a (near) complete response after neoadjuvant treatment [22]. Trials such as STARTREC are exploring neoadjuvant treatment as an alternative to resection for low-risk tumors aiming for organ preservation [23]. This has sparked a growing interest in predicting response at baseline (i.e., before treatment), in addition to assessing post-treatment response. Standardized reporting templates currently do not include provisions for response prediction [7, 19]. The results of our current survey suggest that radiologists are increasingly encouraged to integrate response estimation into their reports, paving the way for more nuanced MDT discussions. However, at the time of writing this paper, the evidence regarding the benefits and clinical impact of pre-treatment response prediction remain unclear.

Despite the positive findings, several challenges were also identified via the questionnaire. Respondents noted inconsistent feedback delivery during and after studies. While some found feedback highly useful, others recommended more personalized and targeted feedback. Additionally, recall bias and positive selection bias may have influenced results, as the questionnaire was distributed long after study completion and participants with positive experiences may have been more likely to respond. Lastly, we acknowledge that our impact evaluation is based on self-reported perceptions without objective measures of behavior change or practice transformation. As such, no firm conclusions can be drawn regrading true clinical impact. As a follow-up to this study, future research should aim to more objectively assess changes in clinical practice, for example by analyzing MRI reports from before and after participation in such validation studies. This could be done in a similar way as in a previous study by Bogveradze et al. who examined evolutions in rectal cancer MRI reporting practices in the Netherlands after the introduction of structured reporting templates for imaging and novel grading systems for lymph node assessment in Dutch guidelines updates [24].

In conclusion, this questionnaire demonstrates that participation in web-based validation studies positively influences self-reported personal and institutional practices in MRI-based (re)staging of rectal cancer. These findings emphasize the crucial role web-based studies can play a role in translating research into practice, fostering adoption of evidence-based diagnostic tools, and promoting international collaboration among radiologists. They also highlight the broader potential of web platforms for research, training, and education. To maximize impact, future studies should ensure consistent feedback and explore integrating training modules directly into the research workflow.

## Electronic supplementary material

Below is the link to the electronic supplementary material.

Supplementary Material 1

Supplementary Material 2

## Data Availability

No datasets were generated or analysed during the current study.
