# Supplementary material for: Advancing MRI diagnostic practices in rectal cancer: exploring the impact of web-based multi-reader study participation
Source: Abdom Radiol (NY). 2025 Jul 28;51(3):1169–76. doi: 10.1007/s00261-025-05104-6 (PMC12971794; doi:10.1007/s00261-025-05104-6)
Supplement: Supplementary file 1 — Supplementary Material 1 [file 261_2025_5104_MOESM1_ESM.pdf]

Supplement 1 – Questionnaire

PART I – GENERAL QUESTIONS

1. **What is your name?**
   …..

2. **What is your gender?**
   ☐ Male
   ☐ Female
   ☐ Unspecified

3. **In what year did you complete your residency training?**
   …..

4. **What was your profession and clinical experience level at the time of your study participation?**
   ☐ Abdominal radiologist with dedicated expertise in (colo)rectal cancer
   ☐ Abdominal radiologist
   ☐ General radiologist
   ☐ Radiology resident

5. **What is your main workplace?**
   ☐ Comprehensive cancer center / Dedicated oncology center
   ☐ Academic hospital
   ☐ Non-academic / General hospital
   ☐ Private practice / Other

6. **In which country do you work?**
   …..

7. **In which of our study projects did you participate (multiple answers allowed)?**
   ☐ A. Project focused on primary staging using structured reporting templates*
   ☐ B. Project focused on baseline response prediction and posttreatment response assessment^
   ☐ C. Project focused on the sigmoid take-off#

      * El Khababi et al. Br J Radiol 2023;96(1150):20230091
      ^ El Khababi et al. Colorectal Dis 2023;25(9):1878-1887; El Khababi et al. Eur Radiol 2023;33(6):4367-4377; El Khababi et al. Abdom Radiol (NY) 2023;48(10):3039-3049
      # Bogveradze et al. Eur J Surg Oncol 2022;48(1):237-244

8. **How would you rate your overall experience with our webplatform (iScore)?**
   ☐ Highly negative
   ☐ Moderately negative
   ☐ Equivocal
   ☐ Moderately positive
   ☐ Highly positive
   → Could you comment on the main positive sides: ……
   → Could you comment on the main negative sides & provide suggestions for improvement: …..

9. **Did you receive any feedback from us on your MRI interpretations during or after you completed your case readings?**
   ☐ No
   ☐ I don't remember
   ☐ Yes, I received a general overview of my scorings compared to the scores of the rest of the group and/or the standard of reference
   ☐ Yes, I received more detailed / personalized feedback
   → If yes, how would you value the quality of the feedback you received?
   ☐ not useful, ☐ slightly useful, ☐ moderately useful, ☐ very useful, ☐ highly useful
   → Do you have any suggestions how we could have improved the quality of our feedback? …..

10. **Did you learn anything from participating in our study/studies and from reading the final study results?**
   ☐ No
   ☐ Slightly
   ☐ Somewhat
   ☐ Much
   ☐ Extremely

11. **To what extent did participation in our study/studies affect your personal reporting practice?**
   ☐ No
   ☐ Slightly
   ☐ Somewhat
   ☐ Much
   ☐ Extremely
   → If you answered positively, could you explain and/or provide us with an example? …..

12. **Did you share and/or discuss the results of our study/studies with any of your colleagues?**
   ☐ No
   ☐ Yes, I shared the publication(s) with one or more colleagues
   ☐ Yes, I shared the publication(s) and actively discussed the findings with my colleague(s) to see

if and how the results could be implemented in our clinical practice

13. **Would you say that your participation had an effect on general clinical practice within your department of institution?**
    ☐ No
    ☐ Slightly
    ☐ Somewhat
    ☐ Much
    ☐ Extremely
    → If your answered positively, could you explain and/or provide us with an example? …..

PART II – STUDY SPECIFIC QUESTIONS

A. Only to be filled in for participants of project A (primary staging using structured reporting templates):

1. **Before participating in this study, did you use a standardized reporting template for rectal cancer staging?**
   ☐ No
   ☐ Yes, I used the ESGAR template
   ☐ Yes, I used another publically available template (e.g., from national society, SAR, RSNA)
   ☐ Yes, I used a template developed within my own institution

2. **Did participation in our study have any effect on this?**
   ☐ No
   ☐ Yes, I adopted the ESGAR template
   ☐ Yes, I adopted a different staging template or incorporated a self-developed template

3. **To what extent did participation in our study (and reading the study results) have any additional effects on the way you interpret and stage rectal cancer and/or the way you communicate your finding in your local MDT?**
   ☐ Not at all
   ☐ Slightly
   ☐ Somewhat
   ☐ Much
   ☐ Extremely
   → If you answered positively, could you provide us with an example? …..

4. **Do you think that based on the outcomes of these studies, any changes should be made to the recommended reporting templates for staging and/or restaging of rectal cancer (such as those**

published by ESGAR)?

☐ No

☐ Possibly → Please explain …..

☐ Yes → Please explain …..

B. Only to be filled in for participants of project B (baseline response prediction and posttreatment response assessment):

1. **Before participating in our study, did you already use the <u>mrTRG</u> to assess response on restaging MRI in your own clinical practice?**

   ☐ No

   ☐ Occasionally

   ☐ Sometimes (e.g., half of the time)

   ☐ Most of the time

   ☐ Always

2. **Do you nowadays apply the <u>mrTRG</u>?**

   ☐ No

   ☐ Occasionally

   ☐ Sometimes (e.g., half of the time)

   ☐ Most of the time

   ☐ Always

3. **Did your participation in our study have any effect on this?**

   ☐ No

   ☐ Yes → Please explain why you decided to change your practice: ……

4. **Before participating in our study, did you already use <u>DWI</u> to assess response on restaging MRI in your own clinical practice?**

   ☐ No

   ☐ Occasionally

   ☐ Sometimes (e.g., half of the time)

   ☐ Most of the time

   ☐ Always

5. **Do you nowadays use <u>DWI</u>?**

   ☐ No

   ☐ Occasionally

   ☐ Sometimes (e.g., half of the time)

☐ Most of the time

☐ Always

6. **Did your participation in our study have any effect on this?**

   ☐ No

   ☐ Yes → Please explain why you decided to change your practice: ……

7. **If you use DWI, how do you apply it?**

   ☐ Not applicable / I do not use DWI

   ☐ I integrate it into my overall assessment, but do not use a specific DWI scoring system

   ☐ I use the modified TRG score

   ☐ I use the DWI pattern-approach described by Lambregts et al.

   ☐ I use a different DWI scoring system

8. **Before participating in our study, did you already use the <u>split scar sign</u> (described by Santiago et al) to assess response on restaging MRI in your own clinical practice?**

   ☐ No

   ☐ Occasionally

   ☐ Sometimes (e.g., half of the time)

   ☐ Most of the time

   ☐ Always

9. **Do you nowadays apply the <u>split scar sign</u>?**

   ☐ No

   ☐ Occasionally

   ☐ Sometimes (e.g., half of the time)

   ☐ Most of the time

   ☐ Always

10. **Did your participation in our study have any effect on this?**

    ☐ No

    ☐ Yes → Please explain why you decided to change your practice: ……

11. **Before participating in our study, did you routinely <u>assign a yT-stage</u> when reporting a restaging MRI after neoadjuvant treatment?**

    ☐ No

    ☐ Occasionally

    ☐ Sometimes (e.g., half of the time)

    ☐ Most of the time

☐ Always

12. Do you nowadays assign a yT-stage?

☐ No

☐ Occasionally

☐ Sometimes (e.g., half of the time)

☐ Most of the time

☐ Always

13. Did your participation in our study have any effect on this?

☐ No

☐ Yes → Please explain why you decided to change your practice: ……

14. Before participating in our study, did you ever include a response estimation/prediction in your baseline staging report (i.e., provide a chance estimation of whether a patient will likely achieve a complete response).

☐ No

☐ Occasionally

☐ Sometimes (e.g., half of the time)

☐ Most of the time

☐ Always

15. Do you nowadays include a response estimation in your baseline report?

☐ No

☐ Occasionally

☐ Sometimes (e.g., half of the time)

☐ Most of the time

☐ Always

16. Do you discuss the chance of achieving organ-preservation in your MDT as part of the treatment decision making process (at the time of baseline staging)?

☐ No

☐ Occasionally

☐ Sometimes (e.g., half of the time)

☐ Most of the time

☐ Always

17. Did your participation in our study have an effect on the way you report and/or discuss this in the MDT?

☐ No

☐ Yes → Please explain why you decided to change your practice: ……

18. **Do you think that based on the outcomes of these studies, any changes should be made to the recommended reporting templates for staging and/or restaging of rectal cancer (such as those published by ESGAR)?**

    ☐ No

    ☐ Possibly → Please explain …..

    ☐ Yes → Please explain …..

C. Only to be filled in for participants of project C (Sigmoid take-off):

1. **Before participation in our study, did you use the STO as a landmark to discern rectal from sigmoid cancer in your own daily practice?**

   ☐ No

   ☐ Occasionally

   ☐ Sometimes (e.g., half of the time)

   ☐ Most of the time

   ☐ Always

2. **Do you nowadays use the STO?**

   ☐ No

   ☐ Occasionally

   ☐ Sometimes (e.g., half of the time)

   ☐ Most of the time

   ☐ Always

3. **Did your participation in our study have any effect on this?**

   ☐ No

   ☐ Yes → Please explain why you decided to change your practice: ……

4. **Do you think that based on the outcomes of this study, any changes should be made to the recommended reporting templates for staging and/or restaging of rectal cancer (such as those published by ESGAR)?**

   ☐ No

   ☐ Possibly → Please explain …..

   ☐ Yes → Please explain …..
