# Supplementary material for: Advancing MRI diagnostic practices in rectal cancer: exploring the impact of web-based multi-reader study participation
Source: Abdom Radiol (NY). 2025 Jul 28;51(3):1169–76. doi: 10.1007/s00261-025-05104-6 (PMC12971794; doi:10.1007/s00261-025-05104-6)
Supplement: Supplementary file 2 — Supplementary Material 2 [file 261_2025_5104_MOESM2_ESM.pdf]

Glossary of reporting tools and diagnostic grading systems investigated in previously published works that were addressed in our questionnaire study:

Terms are listed in alphabetical order:

- *Diffusion-weighted imaging (DWI) patterns*:
  Scoring system introduced by Lambregts et al. to discern complete responders from patients with residual tumor on MRI after neoadjuvant treatment by combining morphological patterns on pre- and post-treatment T2-weighted imaging with distinct DWI signal patterns on post-treatment MRI [1].

- *European Society of Gastrointestinal and Abdominal Imaging (ESGAR) reporting template*:
  Structured reporting templates for the primary staging and restaging of rectal cancer that include tumor location and size, tumor (T) stage, nodal (N) stage, tumor deposits, extramural vascular invasion (EMVI), mesorectal fascia (MRF) and sphincter involvement [2].

- *MRI tumor regression grade (mrTRG)*:
  Grading system to classify the degree of fibrotic transformation versus residual tumor on MRI after neoadjuvant treatment. The mrTRG was compared to other methods of response evaluation in a multi-reader setting by el Khababi et al. [3]
  - mrTRG1 = no/minimal fibrosis
  - mrTRG2 = dense fibrotic scar without macroscopic tumor signal
  - mrTRG3 = fibrosis predominates but there are obvious areas of tumor signal
  - mrTRG4 = tumor signal predominates with little/minimal fibrosis
  - mrTRG5 = tumor signal only, no fibrosis (includes cases with tumor progression)

- *Modified mrTRG*:
  Adaptation of the mrTRG that combines the degree on fibrotic transformation on T2-weighted MRI with the presence or absence of diffusion restriction on DWI [3,4,5].
  - 0 = no tumor signal on T2W MRI, no diffusion restriction (complete regression)
  - 1 = predominant fibrosis on T2W MRI, focal diffusion restriction (intermediate regression)
  - 2 = predominant tumor on T2W MRI, focal or mass-like diffusion-restriction

- *Pre-treatment response prediction:*
  El Khababi et al. evaluated three methods to estimate the likelihood that patients would undergo a complete or near-complete response to neoadjuvant treatment by assessing the morphology and risk profile of the primary tumor on baseline staging MRI [6]. The first method was a 5-point confidence score previously published by van Griethuysen et al. [7] based on a combination of tumor size, signal, shape, T- and N-stage, EMVI and MRF involvement. The second score was a 4-point risk score with 1 point each for high risk T-stage, obvious MRF involvement, obvious nodal involvement, and obvious EMVI. The third score was a 2-point global estimate (unlikely or likely to achieve a (near-)complete response).

- *Sigmoid take-off (STO)*:
  Anatomic landmark to define the boundary between the rectum and sigmoid colon on imaging (typically MRI). The STO is defined as the point from which the sigmoid colon

sweeps horizontally (away from the sacrum) on sagittal views, and centrally on axial views. In the publication by Bogveradze et al. the value of the STO to discern rectal from sigmoid tumors on MRI was tested in a multi-reader setting using multicenter data [8].

- *Split scar:*
  Morphologic sign introduced by Santiago et al. to discern complete responders from patients with residual tumor by assessing the presence or absence of a typical layered appearance of the fibrotic tumor bed ('scar') on MRI after neoadjuvant treatment [9]

- *yT-staging*:
  Evaluation of the tumor stage after neoadjuvant treatment (yT) on MRI. The pros and cons of yT-staging after chemoradiotherapy were addressed in a multireader study by el Khababi et al. [10]

References:

1. Lambregts DMJ, Delli Pizzi A, Lahaye MJ, et al (2018) A pattern-based approach combining tumor morphology on MRI with distinct signal patterns on diffusion-weighted imaging to assess response of rectal tumors after chemoradiotherapy. Dis Colon Rectum 61(3):328–33
2. Beets-Tan RGH, Lambregts DMJ, Maas M, et al (2018) Magnetic resonance imaging for clinical management of rectal cancer: updated recommendations from the 2016 European Society of Gastrointestinal and Abdominal Radiology (ESGAR) consensus meeting. Eur Radiol 28(4):1465–1475
3. El Khababi N, Beets-Tan RGH, Tissier R, et al (2023). Comparison of MRI response evaluation methods in rectal cancer: a multicentre and multireader validation study. *Eur Radiol* 33(8): 4367–4377.
4. Lee MA, Cho SH, Seo AN, et al (2017) Modified 3-point MRI-based tumor regression grade incorporating DWI for locally advanced rectal cancer. AJR Am J Roentgenol 209(6):1247–1255
5. Haak HE, Maas M, Lahaye MJ, et al (2020) Selection of patients for organ preservation after chemoradiotherapy: MRI identifies poor responders who can go straight to surgery. Ann Surg Oncol 27(8):2732–2739
6. El Khababi N, Beets-Tan RGH, Tissier R, et al (2023). Predicting response to chemoradiotherapy in rectal cancer via visual morphologic assessment and staging on baseline MRI: a multicenter and multireader study. Abdom Radiol (NY) 48(10): 3039–3049
7. van Griethuysen JJM, Lambregts DMJ, Trebeschi S, et al (2020) Radiomics performs comparable to morphologic assessment by expert radiologists for prediction of response to neoadjuvant chemoradiotherapy on baseline staging MRI in rectal cancer. Abdom Radiol (NY) 45(3):632-643
8. Bogveradze N, Lambregts DMJ, El Khababi N, et al (2022). The sigmoid take-off as a landmark to distinguish rectal from sigmoid tumours on MRI: reproducibility, pitfalls and potential impact on treatment stratification. Eur J Surg Oncol 48(1): 237–244
9. Santiago I, Barata M, Figueiredo N, et al (2020). The split scar sign as an indicator of sustained complete response after neoadjuvant therapy in rectal cancer. Eur Radiol 30(1): 224–238
10. El Khababi N, Beets-Tan RGH, Tissier R, et al (2023). Sense and nonsense of yT-staging on MRI after chemoradiotherapy in rectal cancer. Colorectal Dis 25(9): 1878–1887.
